# Influence of Selected Factors of Vibratory Work Hardening Machining on the Properties of CuZn30 Brass

**DOI:** 10.3390/ma17235913

**Published:** 2024-12-03

**Authors:** Damian Bańkowski, Anna Kiljan, Irena M. Hlaváčová, Piotr Młynarczyk

**Affiliations:** 1 Department of Materials Science and Materials Technology, Faculty of Machatronics and Mechanical Engineering, Kielce University of Technology, al. 1000-lecia P.P. 7, 25-314 Kielce, Poland; piotrm@tu.kielce.pl; 2 Department of Engineering and Biomedical Materials, Faculty of Mechanical Engineering, Silesian University of Technology, Akademicka 2A, 44-100 Gliwice, Poland; anna.kiljan@polsl.pl; 3 Department of Physics, Faculty of Electrical Engineering and Computer Science, VSB–Technical University of Ostrava, 17. listopadu 2172/15, Poruba, 70800 Ostrava, Czech Republic; ikki.imkjh@gmail.com

**Keywords:** vibratory machining, work hardening, brass, recrystallization, crushing

## Abstract

The purpose of this study was to determine the effect of selected vibratory strengthening machining factors on the properties of CuZn30 brass. Vibratory strengthening machining was carried out using metal media dedicated to polishing processes, which also contributed to strengthening the treated surfaces. The test samples were cut with an abrasive water jet and recrystallized to obtain a soft microstructure. An orthogonal, two-factor five-level plan was used for the study. The effect of vibration frequency and vibratory machining time on selected changes in parameters of the geometric structure of the surface and hardness of the surface layer was determined using Statistica software version 10 (64-bit). Higher vibration frequencies for vibratory machining increased the hardness of machined surfaces by as much as 50 HV_0.02._ The arithmetic mean deviation of the height of surface irregularities from the reference plane, Sa, decreases with increasing the time of vibratory machining. A value of Sa = 0.168 µm was obtained after 87 min of consolidation, compared to an initial surface of S_a_ = 0.65 µm.

## 1. Introduction

According to the literature [1,2,3,4], vibratory machining is defined as a type of mechanical treatment, although one can also find a definition of vibratory machining as a type of chemical-mechanical treatment [5,6,7]. Dedicated cutting fluids or abrasive pastes are then used as the chemical agent. Synonyms for vibratory machining include “vibrational-abrasive machining” [8], roto-finish, vibro-abrasive machining, vibratory polishing, etc.

The use of vibratory motion allows us to distinguish this type of container machine tool from rotary machines. The operator can precisely set the vibration frequency of the device by controlling the engine speed. Some machine tools have an additional option to adjust the vibration amplitude. This is usually achieved by using additional mass on the motor shaft or through the angular adjustment of the masses mounted on the motor shaft.

Vibration machining is one of the methods of using the mechanical effects of loose media for surface treatment. The media and workpieces placed in the work tank are subject to mutual impact. The use of vibratory movement (vibration) in comparison to rotary machines translates into faster results. In Reference [9], Marciniak presented how the type and time of container processing affect the changes in the arithmetic mean of surface roughness, R_a_. Reference [9] shows that extending the time of vibratory machining above a certain value does not cause a decrease in surface roughness parameters. By appropriately selecting media–abrasive media and their application to the process, abrasive machining is possible. It is possible to remove corrosion, oxide layers, paint coating residues, and to deburr surfaces for further processing. A wide range of media dedicated to container processing also allows for polishing and brightening surfaces. A special case is the use of media in the form of steel balls, which smooth and strengthen the surface. Moreover, abrasive pastes or working fluids can be additionally used for container processing. They are designed to support the processes carried out, to achieve faster surface polishing, to remove material erosion products, or to protect surfaces against possible oxidation and corrosion. In order to obtain a high-gloss effect, the authors suggest two-stage processing [10] or three-stage grinding, lapping, and polishing [11]. In [10], Bańkowski studied the effect of processing time on changes in the parameters of surface geometric structure, hardness changes, and mass loss. The aim of this study is to extend the existing research by assessing the effect of vibration frequency. The obtained results will allow for the determination of the effect of machining time and frequency of vibration processing on changes in the surface geometric structure and the hardness of the constituted layer.

Of particular interest are the changes in the surface layer of soft materials subjected to cold plastic deformation (below the recrystallization temperature). Plastic deformations occurring during the impact of loose metal media on the workpieces cause a significant increase in the number of crystal lattice defects, mainly point and linear ones [12]. This translates into the accumulation of deformation energy [13], which is higher the lower the vibratory work hardening temperature is. This causes changes in density and changes in properties observed primarily as an increase in hardness and strength and a decrease in plasticity [12,13,14,15,16]. In favorable conditions, even more than 10% of the energy used for deformation can be stored in the form of structural defects. [12]. It is the dislocations accumulated in the material that are responsible for mechanical strengthening, which is manifested by an increase in strength properties—hardness, yield strength, and durability—and a decrease in plastic properties, such as impact strength, elongation, and narrowing [17]. Increasing the density of dislocations causes the strengthening of metals and alloys because the distance between dislocations decreases. At the same time, the forces of interaction between them increase. This causes the movement of the dislocations to be blocked, resulting in greater and greater stress being necessary for further deformation [12].

**Figure 1:**
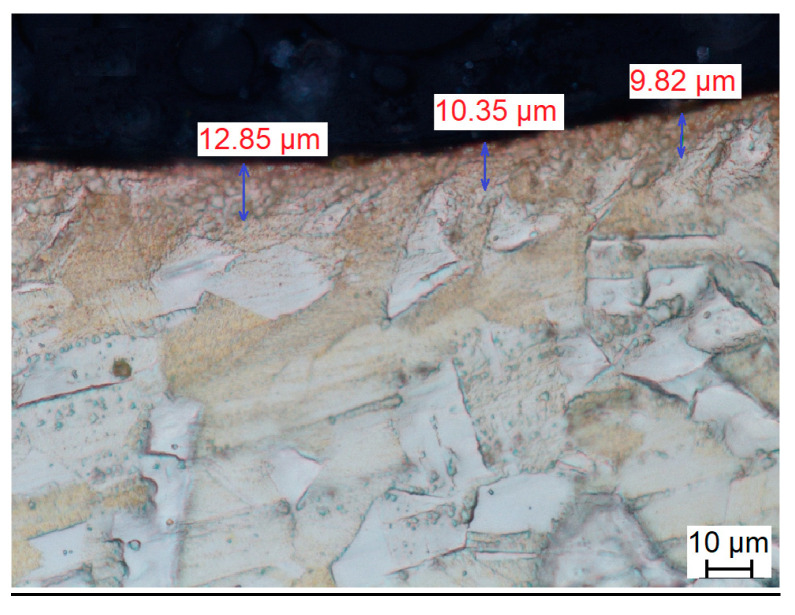
Microphotographs of brass (CuZn30) after plastic working, annealing and vibratory work hardening.

Work hardening causes the grains to lengthen and thus increases the number of dislocations in the material. The depth of the occurring changes is determined by the value of the impact energy of workpieces and tools. Based on the observations made, the greatest changes can be observed at the very contact surface of the material with the tools (steel media). As the distance from the surface increases, the observed changes decrease until they disappear completely. Examples of brass microstructure changes can be observed in Figure 1.

The introduction of cold work causes microstructure changes, which results in changes in grain size. With increasing distance from the processing surface, grain deformation decreases, and a large number of annealing twins are visible.

Brass CuZn30 (so-called scale brass) is a two-component alloy composed of copper and zinc. Produced for thousands of years, it is valued for its workability, hardness, corrosion resistance, and attractive appearance [18]. CuZn30 brass has high plasticity and susceptibility to cold working and good strength properties [19]. It is used to make the cores and housings of radiators, tanks, projectiles, seals, lamps, bowls, trays, metal haberdashery elements, components for power engineering and electronics, and drawing elements [20].

Changing the properties of the surface layer of brasses, especially cartridge case brasses, can be interesting. Due to the material used to produce the cartridge case, repeatability of the vibration machining process is required. Obtaining finished products, especially those with complex geometry characterized by a soft core and a crushed, hard top layer, can be useful in special applications.

The properties of brasses subjected to cold work and heat treatment have been the subject of numerous studies. In Broniewski’s work [21], one can learn that “the cold work of brass has been the subject of more studies than the cold work of any other alloy”. With the increase in the degree of deformation, the strength properties increase (Rm-breaking strength limit), as does the elasticity limit, E [21]. Moreover, with the increase in the degree of deformation, the plastic properties decrease, i.e., elongation (A, %) or narrowing (Z, %). Therefore, by introducing cold work to the surface layer, it is possible to harden and improve the properties of elements made of brass. One of the potential strengthening methods is burnishing. One variety of dynamic burnishing is shot peening or vibratory machining [22]. The impact of strengthening tools causes a change in the properties of the formed surface layer, which is manifested in particular by an increase in hardness [23,24,25,26]. This change in hardness causes a change in residual stresses and changes in the microstructure of the copper. Deformations occur as a result of stresses exceeding the limits of proportionality, elasticity, and plasticity [27]. As reported in [27], plastic deformation and the accompanying work hardening allows the hardness of Hadfield steel to increase from 225 HV_5_ up to 750 HV_5_.

Reference [10] demonstrated the beneficial effect of vibration treatment on reducing the values of the geometric structure parameters of brass surfaces. Even as Bechciński [28] mentions, the deliberate introduction of vibrations to the classical grinding process and control of these vibrations can result in improvement of the parameters of the geometric surface structure (SGP). In the work of Liu [29], the influence of air pressure and process duration on the surface roughness of a titanium alloy was studied.

Shiu [30] studied the effects of shot peening and vibratory machining on the fatigue strength of TC17 alloy. He showed that the copper’s fatigue strength after shot peening and vibratory machining was higher than that of shot peening alone. This is due to the fact that shot peening and vibratory machining can result in lower surface roughness and a relatively stable gradient microstructure, as well as a layer of residual compressive stresses compared to the surface after shot peening [30].

In the work of Matuszak [31], it was shown that the vibration amplitude has an effect on the hardness and thickness of the strengthened layer of a titanium alloy. It was shown that the amplitude has a greater effect on the increase in the hardening of the surface layer in comparison with the duration of the treatment. The greatest thickness of the hardened layer, exceeding 200 µm, was obtained after shot blasting with an amplitude A = 60 mm.

Obtaining improved material properties on the surface and a ductile core at the same time may allow for reducing the mass of elements and structures, while reducing the dimensions of cross-sections [32]. The results of the study can be used to optimize the design of vibration or strengthening machining processes.

## 2. Materials and Methods

In order to assess the impact of vibratory strengthening machining, it was decided to perform tests on a soft material. For the tests, samples of CuZn30 brass were prepared in the form of cuboids with dimensions of 30 mm × 12 mm and 3 mm. Their chemical composition is given in Table 1.

In order to limit the temperature introduced into the material during its separation, high-pressure abrasive water jet cutting (AWJ) was used on the PTV WJ1020-1Z-EKO machine (PTV s.r.o., Hostivice, Czech Republic). In [33], the authors proved that, for S235JR steel, at distances above 150 µm from the cutting surface, there is no significant effect of temperature on the material.

Recrystallization annealing is performed to eliminate the effects of cold working [12]. Therefore, in order to obtain a soft microstructure that can be strengthened by vibratory hardening (polishing) machining, the samples were previously annealed. The samples were annealed at 580 °C for 60 min and cooled in water. Then, the samples were subjected to vibratory hardening machining. The Rollwasch SMD-25-R vibration machine (Rollwasch Italiana S.p.a., Albiate (MB), Italy) with a tumbler volume of 25 dm^3^ and a vibration frequency range of 30 to 50 Hz was used for the tests. The samples were subjected to a strengthening treatment by vibratory machining using round steel balls-SB 3.1 lotto (Rollwasch Italiana S.p.a., Albiate (MB), Italy) with a diameter of 3.1 mm and the addition of approx. 200 mL of FE L120 B32/R fluid (Rollwasch Italiana S.p.a., Albiate (MB), Italy) with antioxidant properties and ensuring effective smoothing and polishing. The filling level of the working tank-tumbler was set to 40%.

**Figure 2:**
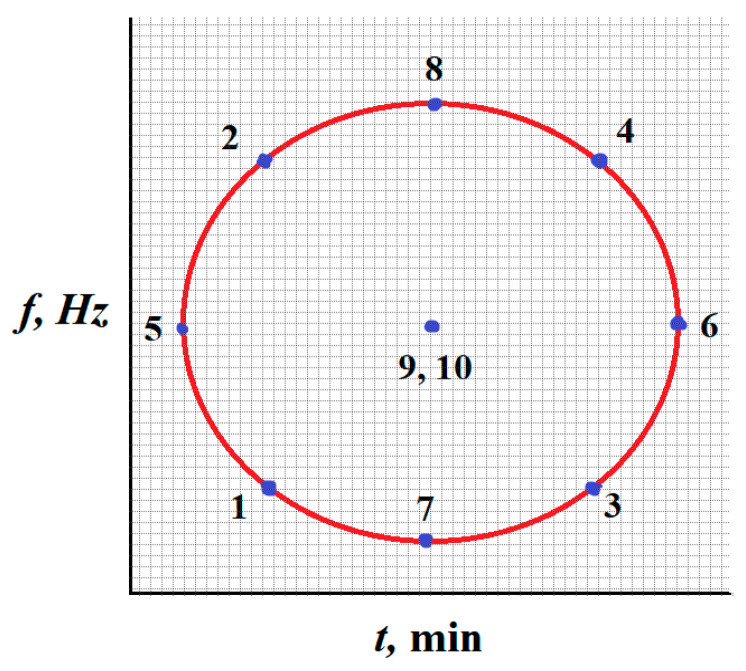
Location of test points (systems in the adopted plan).

The research was carried out according to a compositional plan for two parameters with five levels of variability, as shown in Figure 2. The planned experiment was carried out using Statistica 10 64-bit software (TIBCO Software Inc., Tulsa, OK, USA). A five-level design was chosen in order to examine the influence of independent variables on the output factors to a greater range than is possible with a three-factor design [34].

The process variation intervals were selected for:

- processing time, t, which took on the values of 3, 15, 45, 75, and 87 min, which correspond to the code values: −1.414; −1; 0; 1; and 1.414 vibration frequency, f, of the working tank, which took on the values of 30.6, 33.3, 40, 46.7, and 49.4 Hz, which correspond to the code values: −1.414; −1; 0; 1; and 1.414.

## 3. Results and Discussion

This study analyzed changes in the parameters of the surface geometric structure and the hardness of the surface layer of a copper alloy. The 3D surface roughness parameters were determined using a non-contact 3D Taylor–Hobson Talysurf CCI Lite profilometer (Taylor–Hobson, Leicester, UK). Surfaces of 1.6 × 1.6 mm from the middle part of the samples were analyzed. A 0.8 mm Gaussian filter was used to calculate the amplitude and height indices of the geometric surface structure.

In order to describe the geometric structure of the surface after vibratory machining, the following parameters were considered: the arithmetic mean deviations of the surface unevenness height from the reference plane, S_a_, S_p_, the maximum surface peak height, S_z_, the maximum surface height understood as the difference between the maximum height of the surface peak and the maximum height of the surface recess (S_z_ = S_p_ − S_V_), S_sk_, the kurtosis of the surface, and S_ku_, the surface asymmetry [35,36,37].

**Figure 3:**
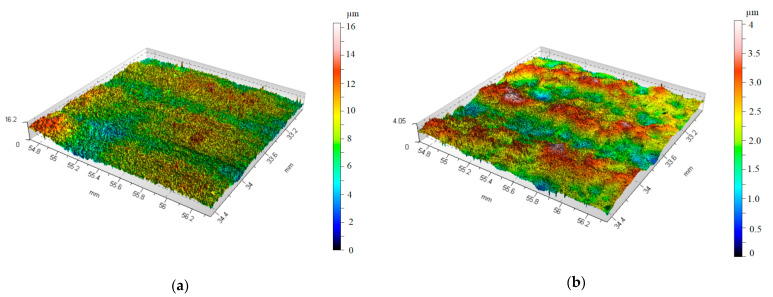
Isometric views of the measured surfaces: (**a**) after softening heat treatment; (**b**) after 87 min of vibration hardening treatment.

Figure 3a shows the surface of the sample after softening heat treatment, and Figure 3b shows the surface after 87 min of vibratory work hardening machining. The hardness of the CuZn30 brass was measured at a load of 20 g using an Innovatest Nexus 400 tester (Innovatest, Maastricht, The Netherlands). The hardness values given in Table 2 are the averages of at least 4 measurements.

Based on the data obtained from the conducted experimental studies, mathematical models of the vibratory machining process were generated using Statistica software. The mathematical relationships between the values of the input quantities and the values of the tested quantities were possible to express thanks to the use of the Response Surface Methodology (RSM) method. The results were evaluated using analysis of variance (ANOVA). For each equation, the coefficient of determination, R^2^, and the alternative coefficient of determination, R^2^-Adj, were determined. The adjusted version of the R^2^ coefficient takes into account the number of variables in the model and thus allows for a more conservative assessment of the fit. The ANOVA results for the assumed significance level of 0.05 are presented in Table 3 and Table 4. In the final stage, the prediction models were evaluated [38].

Multiple regression with backward elimination (for the assumed significance level α = 0.05) was used to describe the response function as a second-degree polynomial. The homogeneity of variance was checked for the significance level of α = 0.05. Conclusions about the significance or insignificance of the influence of a specific term of the regression equation were made. The terms that were not significant from the point of view of statistical inference were rejected, and only the significant terms (P below 0.05) were taken into account. In the case of lack of homogeneity of variance for all terms of the equation, searching for the function of the research object is impossible. It can then be stated that, for the assumed significance level α, there are no statistical dependencies of input factors on the tested output feature [39,40,41,42].

The analysis of the influence of individual input factors of the vibro-abrasive machining process on the variance of the HV and S_a_ models indicates that the arithmetic mean deviation of the surface unevenness height from the reference plane after vibratory machining is influenced by the machining time. On the other hand, the hardness of the surface layer is influenced by the vibratory machining frequency. The remaining terms and their mutual interactions (for the assumed significance level α = 0.05) are insignificant.

The determined forms of the hardness equations and the arithmetic mean deviation of the surface unevenness height from the reference plane, as well as the correlation and determination coefficients and the corrected determination coefficient, are given in Table 5.

**Figure 4:**
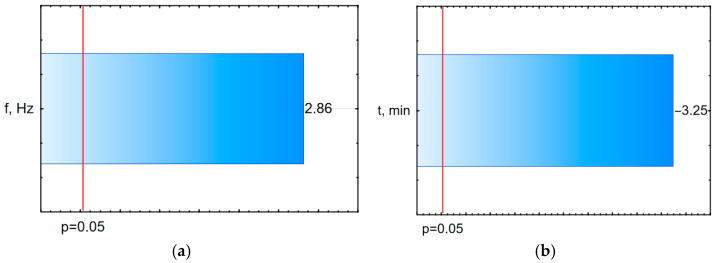
Pareto chart for vibration machining (**a**) HV; (**b**) S_a_.

The developed equations will allow users of vibratory machining devices to determine which input factors of the vibratory machining process should be changed to obtain the desired surface roughness or hardness. So far, there are no mathematical models describing the effect of time and frequency of vibratory machining on HV or SGP parameters. The obtained equations are characterized by a moderately high degree of correlation, R. The calculated percentage influences of individual input factors of the vibratory machining process on the variance of the S_a_ model indicate that the greatest influence on the geometric structure indices of the surface after vibration machining is exerted by the machining time. The hardness of the surface layer is influenced by the frequency of the vibration machining. The remaining terms and their mutual interactions (for the assumed significance level of α = 0.05) are insignificant. This is confirmed by the Pareto charts, which show, in Figure 4, the values of the standardized influence of individual terms of the regression equation on the tested model.

Pareto charts indicate the values of the standardized impact of the terms of the regression equation for the tested models.

The correctness of the developed models was verified by analyzing the residuals. The residual is defined as the difference between the experimental value and the RSM model value [43]. Residual values equal to or close to zero indicate a correctly developed model [44,45]. When the residual values reach values on the order of several dozen percent of the experimentally obtained values, this indicates an incorrectly developed model.

**Figure 5:**
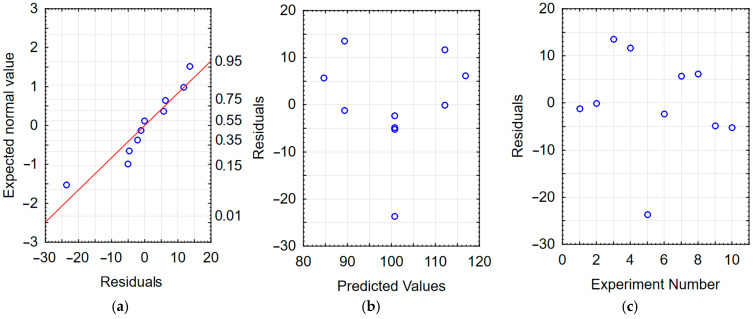
Residual analysis for the HV model of vibratory work hardening machining (**a**) normal plot of residuals; (**b**) residuals relative to predicted values; (**c**) residuals relative to the order of the experiment performed.

**Figure 6:**
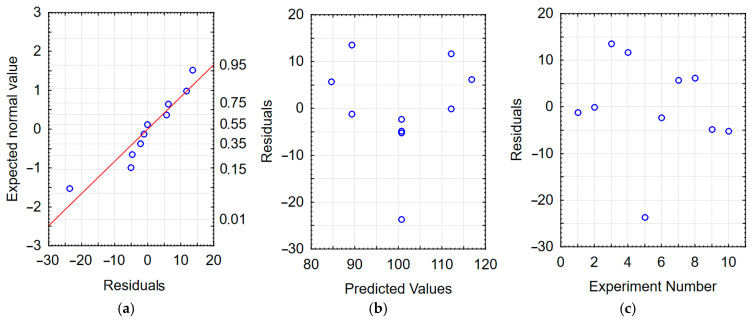
Residual analysis for the S_a_ model of vibratory work hardening machining (**a**) normal plot of residuals; (**b**) residuals relative to predicted values; (**c**) residuals relative to the order of the experiment performed.

Checking the graphs of the dependence of the expected value on the residuals—Figure 5a and Figure 6a—reveals a linear dependence, which indicates that the residuals had a normal distribution; this indicates a good model of the function of the dependence on the machining time and the machining frequency. The dependence of the residuals as a function of the predicted values (from the model developed with RMS) presented in Figure 5b and Figure 6b indicates the random nature of the residuals. Moreover, based on Figure 5c and Figure 6c, it can be stated that the dependence of the residuals on the order of the tests performed is random. The analyses carried out confirm the adequacy of the developed regression models.

**Figure 7:**
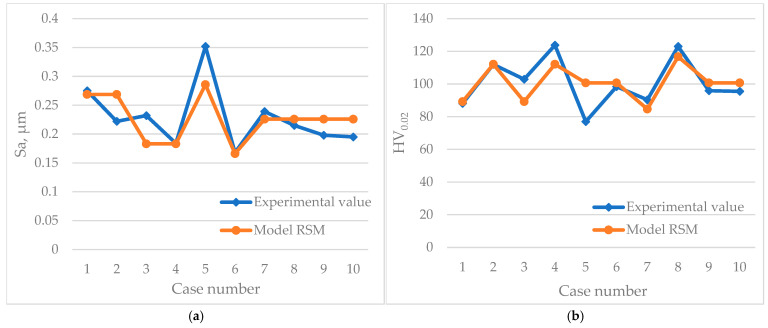
Plots of the experimental value and model RSM: (**a**) S_a_; (**b**) HV_0.02_.

Coefficient R does not graphically represent how closely the accuracy of the models compares with the experimental data. For this reason, in addition to these analyses, the experimental value and the predicted model value are plotted in Figure 7.

Based on the graphs comparing the data collected during the experiments and obtained from the developed RSM models, it can be stated that most of the cases overlap. However, there were single fitting errors for the S_a_ parameter not exceeding 21% for the second and third case numbers (Figure 7a). For the hardness, HV_0.02_, the maximum error reached 31% for the fifth case number (Figure 7b).

**Figure 8:**
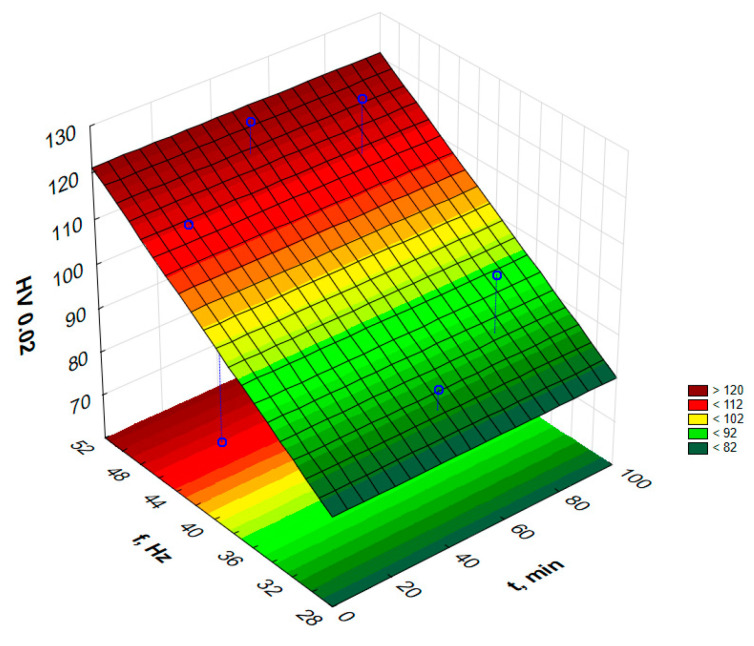
Estimated hardness changes as a function of frequency and time of vibratory work hardening machining.

**Figure 9:**
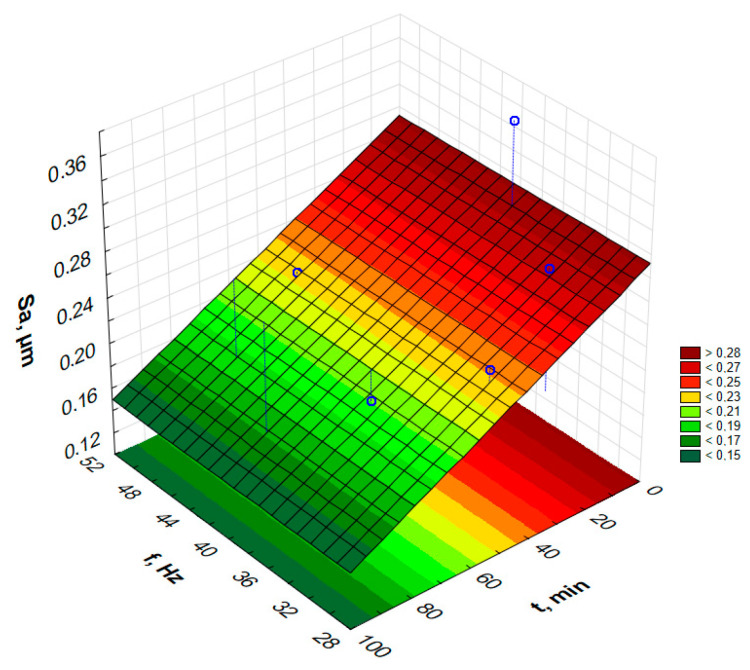
Estimated S_a_ changes as a function of frequency and time of vibratory work hardening machining.

In order to graphically illustrate the dependence of input factors on individual indicators of vibro-abrasive hardening processing, surface fittings in the Statistica program were used. They allowed for the preparation of graphs of the dependence of individual indicators of vibro-abrasive processing on the duration of processing and the vibration frequency of the working container, as shown in —Figure 8 and Figure 9.

The average hardness of the samples in the initial state, before the hardening treatment, was 69.8 HV_0.02_. Based on the graph, it can be concluded that the use of higher frequencies allows for an increase in hardness. The highest hardness of 123.8 HV_0.02_ was obtained a vibration frequency of 49.4 Hz and a treatment time of 45 min. The lowest hardness was the hardness of the sample treated for 3 min at a frequency of 40 Hz, which was 77 HV_0.02_. The higher the vibration frequency, the greater the number of impacts per unit of time. Then, steel balls hit the workpieces, but also objects hit objects or objects hit the walls of the device. Low frequencies mean a reduction in the number of collisions per unit of time. Therefore, statistically, there are fewer collisions per unit of time and the surfaces are characterized by lower hardness than at high frequencies on the order of 50 Hz.

Based on the measurements and the graph of the arithmetic mean deviation of the surface unevenness height from the reference plane as a function of the frequency and duration of the strengthening vibration treatment, it can be seen that the surface without treatment is characterized by the value of the parameter S_a_ = 0.65 µm. After 87 min and 49.4 Hz, it decreased 4 times to 0.168 µm. It can be read from the graph that the extension of the treatment time causes a decrease in the arithmetic mean surface roughness. Based on Figure 9, it can be seen that the lowest values of the parameter S_a_ can be achieved using a long treatment time, i.e., 100 min. The vibration frequency does not affect the changes in the parameter Sa for the same treatment times.

Attempts to determine the dependency function model for the parameters S_p_, S_z_, S_sk_ or S_ku_ do not allow for determining the models for the assumed confidence level α = 0.05. However, the paper presents response graphs for the collected data in Table 2.

**Figure 10:**
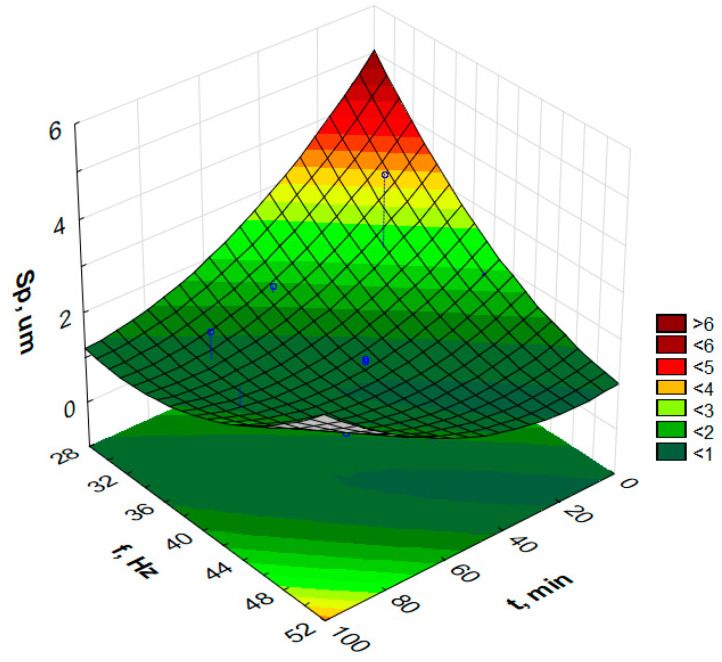
Estimated S_p_ changes as a function of frequency and time of vibratory work hardening machining.

**Figure 11:**
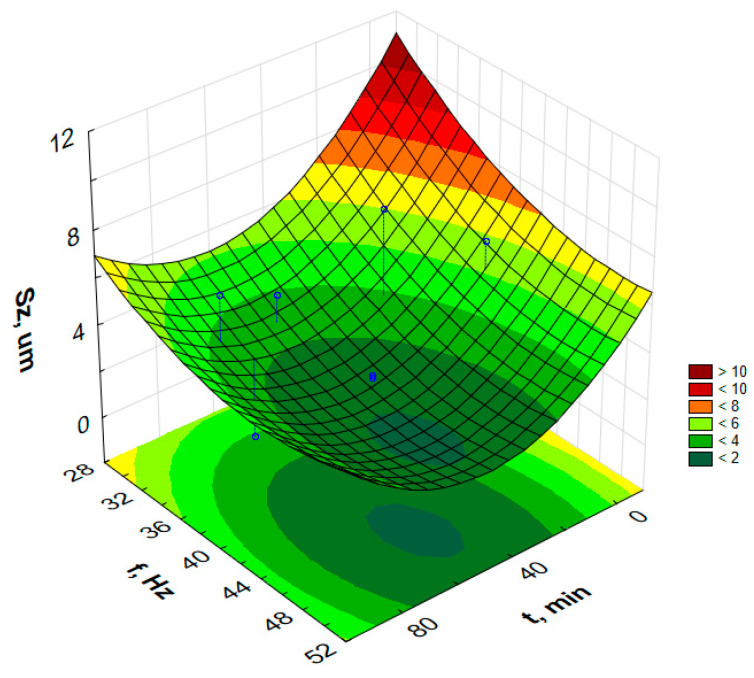
Estimated S_z_ changes as a function of frequency and time of vibratory work hardening machining.

**Figure 12:**
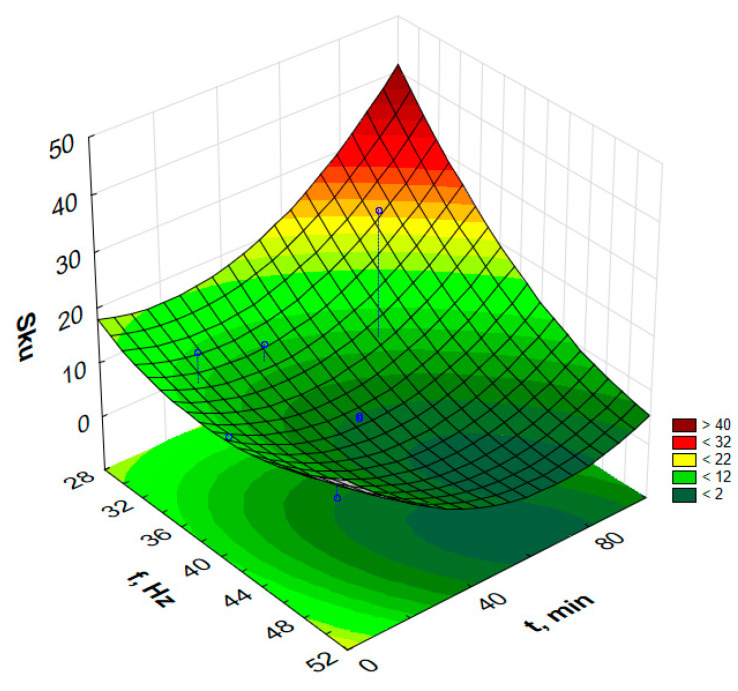
Estimated S_ku_ changes as a function of frequency and time of vibratory work hardening machining.

**Figure 13:**
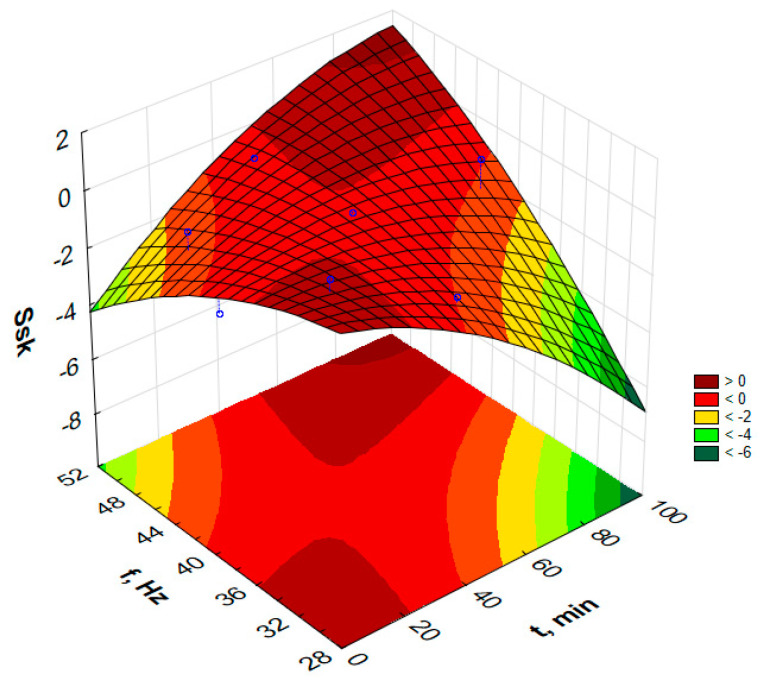
Estimated S_sk_ changes as a function of frequency and time of vibratory work hardening machining.

Figure 10, Figure 11, Figure 12 and Figure 13 present the response surfaces (RSM) obtained from the Statistica program as a function of the frequency and time duration of the treatment. These surface models take into account all terms of the equation (*t*, *t*^2^, *f*, *f*^2^, *tf*).

Based on Table 2 and the prepared S_p_ graph of the highest surface height from the frequency and duration of processing, it can be observed that the surface after annealing and without vibratory machining was characterized by a S_p_ parameter value of over 4 µm. With the duration of vibration processing, the S_p_ parameter significantly decreases to about 1 µm. Then, the surface is smoothed from the elevations on the surface. Longer processing times result in a greater smoothing of the surface. Processing media and workpieces placed loosely in the container allow for free impacts and the crushing of individual ‘hills’ on the surface. The vibration frequency also has a similar effect. Up to about 30 min of vibratory work hardening machining, it can be observed that with the increase in vibration frequency, the S_p_ parameter value decreases rapidly. When extending the processing time beyond 50–60 min, these changes are no longer as spectacular, and one can even be tempted to say that for processing times above 60 min, the frequency does not play a significant role in the changes in the S_p_ parameter.

The character of the S_z_ graph as a function of time and frequency of vibration treatment resembles Figure 10. This is directly related to changes in the S_p_ parameter. Since S_z_ = S_p_ − S_v_ [34], vibration treatment, which causes a change in the S_p_ parameter values, also directly affects the changes in the S_z_ parameter. According to their definition, the S_v_ parameter values are the depths of the geometric structure of the surface. Vibration treatment with steel media in the form of balls does not cause scratching and abrasion of the surface layer, but rather smoothing and leveling of the height of the highest peaks on the surface. The difference between the characters of the graphs that can be observed in the S_z_ graph as a function of frequency and treatment time in comparison to the S_p_ relationship is an increase in the S_z_ parameter value for low treatment frequencies and long treatment times. This may result from errors and possible deep scratches, created, for example, during storage, where the sample could have been rubbed against another sample, or during the vibration treatment itself.

Analyzing the isometric views of the surface topography in Figure 3a,b, it can be seen that the surfaces after vibration machining are more homogeneous (compared to the surfaces without vibratory machining). As can be read from Figure 12, the kurtosis parameter S_ku_ > 3 was obtained for the surface, which means surfaces with many sharp peaks, but the heights of these peaks are low. This is confirmed by the obtained values of height parameters such as S_z_, as shown in Table 2. Based on Figure 12, it can be concluded that reducing the machining frequency causes an increase in the kurtosis parameter, S_ku_. The highest values of S_ku_ parameters in the graph occur for low frequencies (30 Hz) and long machining time (100 min).

Analyzing the skewness parameter (asymmetry coefficient), i.e., S_sk_, revealed negative values for 9 out of 10 tested surfaces. The S_sk_ parameter informs about the nature of the surface and the degree of rounding of the peaks of the elevations [35]. Negative values of the S_sk_ parameter indicate a plateau-like shape of the surface. This is caused by smoothing the largest surface peaks by the action of loose processing media in vibration processing. The surfaces of samples processed at a low frequency and over a long time are characterized by the smallest negative value of the S_sk_ parameter, which means the occurrence of plateau areas and elevations with gentle slopes and rounded peaks on the tested surface [35].

## 4. Conclusions

This research allowed for the determination of mathematical models of changes in hardness and the average arithmetic deviation of the surface unevenness height from the reference plane S_a_ as a function of the duration of vibration treatment and frequency. These equations are characterized by a high value of the correlation coefficient *R* for the assumed significance level α = 0.05.

1. Based on this research, the following conclusions can be drawn:
2. Vibratory work hardening machining causes the strengthening of the microstructure. This is manifested by an increase in the hardness of the samples without treatment, which were characterized by a hardness of approx. 70 HV_0.02_ and, after 87 min of treatment, approx. 120 HV_0.02_.
3. Frequency has a much greater effect on the surface hardness than the time of vibration treatment does.
4. Vibratory work hardening machining (using metal ball media) causes a decrease in the arithmetic mean surface roughness, S_a_, from 0.65 µm to 0.17 µm.

Treatment time has a much greater effect on the decrease in the arithmetic mean surface roughness, S_a_, than the frequency of vibration treatment.

Introduction of strain in the surface layer can be crucial in many applications, allowing for significant savings in the finishing of elements, especially those with complex geometries.

## Figures and Tables

**Table 1:** Chemical composition of CuZn30.

No. Point	Cu, wt.%	Zn, wt. %
1	70.66	29.34
2	70.19	29.81
3	70.65	29.35
mean	70.50	29.50

**Table 2:** Results of hardness and geometric structure parameter measurements of the surface.

No. Experiment	*t*, min	*f*, Hz	HV_0.02_	S_a_, µm	S_p_, µm	S_z_, µm	S_sk_	S_ku_
1	15	33.3	88.10	0.275	3.41	6.16	0.688	14.8
2	15	46.7	112.00	0.222	1.14	2.69	−0.858	6.06
3	75	33.3	102.90	0.232	1.62	5.51	−3.24	26.4
4	75	46.7	123.80	0.184	1.43	2.88	−0.235	4.78
5	3	40	77.00	0.352	1.77	6.01	−1.52	11.3
6	87	40	98.40	0.168	1.03	2.12	−0.0779	4.07
7	45	30.6	90.30	0.239	1.4	3.18	−0.706	5.52
8	45	49.4	123.00	0.215	1.23	2.52	−0.289	3.89
9	45	40	95.90	0.198	1.13	2.35	−0.0314	3.6

**Table 3:** Results of ANOVA-HV analysis for vibratory work hardening treatment.

	Sum of Squares (SS)	Number of Degrees of Freedom	Mean Square	F	P	Influence %
Model	1036.00	1	1036.00	8.19	0.021	
*f*	1036.12	1	1036.12	8.20	0.021	100
Error	1011.29	8	126.4			
Total SS	2047.41	9	R^2^ = 0.51			R^2^-Adj = 0.44

**Table 4:** Results of ANOVA–Sa analysis for vibratory work hardening treatment.

	Sum of Squares (SS)	Number of Degrees of Freedom	Mean Square	F	P	Influence %
Model	0.0145	1	0.0145	10.55	0.012	
*t*	0.0145	1	0.0145	10.55	0.012	100
Error	0.011	8	0.0014			
Total SS	0.025	9	R^2^ = 0.57			R^2^-Adj = 0.51

**Table 5:** Developed regression equations of vibration hardening treatment indices for brass.

The Regression Equation	R	R^2^	R^2^-Adj
HV=32.48+1.705×f	0.71	0.51	0.44
Sa=0.29−0.001425×t	0.75	0.57	0.51

## Data Availability

Data is contained within the article or Supplementary Material.

## Supplementary Materials

The following supporting information can be downloaded at: https://www.mdpi.com/article/10.3390/ma17235913/s1, The methodology of the statistical analysis conducted has been described; Table S1: Dependence of the output parameter as a function of input factors with respect to the correlation coefficient R; Table S2: Results of ANOVA–Sp analysis for vibratory work hardening (without elimination); Table S3: Results of ANOVA–Sz analysis for vibratory work hardening (without elimination); Table S4: Results of ANOVA–Sku analysis for vibratory work hardening (without elimination); Table S5: Results of ANOVA–Ssk analysis for vibratory work hardening (without elimination); Table S6: Regression equations of vibration hardening treatment indices for brass developed (without elimination) [38,40,42,43,44,45,46,47,48,49].
