# Supplementary material for: Influence of Selected Factors of Vibratory Work Hardening Machining on the Properties of CuZn30 Brass
Source: Materials (Basel). 2024 Dec 3;17(23):5913. doi: 10.3390/ma17235913 (PMC11643806; doi:10.3390/ma17235913)
Supplement: Supplementary file 1 [file materials-17-05913-s001.zip › materials-3275836-supplementary.pdf]

materials

MDPI

Article

Influence of Selected Factors of Vibratory Work Hardening Machining on the Properties of CuZn30 Brass

Damian Bańkowski [1,*], Anna Kiljan [2], Irena M. Hlaváčová [3] and Piotr Młynarczyk [1]

[1] Department of Materials Science and Materials Technology, Faculty of Machatronics and Mechanical Engineering, Kielce University of Technology, al. 1000-lecia P.P. 7, 25-314 Kielce, Poland; piotrm@tu.kielce.pl
[2] Department of Engineering and Biomedical Materials, Faculty of Mechanical Engineering, Silesian University of Technology, Akademicka 2A, 44-100 Gliwice, Poland; anna.kiljan@polsl.pl
[3] Department of Physics, Faculty of Electrical Engineering and Computer Science, VSB–Technical University of Ostrava, 17. listopadu 2172/15, Poruba, 70800 Ostrava, Czech Republic; ikki.imkjh@gmail.com
* Correspondence: dbankowski@tu.kielce.pl

Citation: Bańkowski, D.; Kiljan, A.; Hlaváčová, I.M.; Młynarczyk, P. Influence of Selected Factors of Vibratory Work Hardening Machining on the Properties of CuZn30 Brass. *Materials* **2024**, *17*, 5913. https://doi.org/10.3390/ma17235913

Academic Editor: Thomas Niendorf

Received: 9 October 2024
Revised: 25 November 2024
Accepted: 29 November 2024
Published: 3 December 2024

Copyright: © 2024 by the authors. Licensee MDPI, Basel, Switzerland. This article is an open access article distributed under the terms and conditions of the Creative Commons Attribution (CC BY) license (https://creativecommons.org/licenses/by/4.0/).

Based on the conducted experimental studies and obtained data, mathematical models of the vibratory work hardening machining process were generated using Statistica software by applying the Respose Surface Methodology – RSM [41, 44]. RSM was used to determine the mathematical models of the vibratory machining process – the effect of time and frequency of vibrations on the tested dependent variable.

Multiple regression with backward elimination for the assumed significance level of α=0.05 was used to determine the response function [47] in the form of a second-degree polynomial

$$Y = \delta_0 + \delta_1 t + \delta_2 t^2 + \delta_3 f + \delta_4 f^2 + \delta_5 tf + \Delta$$

where:
δ_1 – constant,
f – vibration frequency,
t – machining time,
δ_i – linear, quadratic or interaction coefficient,
Δ – fitting error.

Regression with backward elimination consists in rejecting the equation terms whose p value is greater than 0.05 and re-verifying ANOVA [39, 43, 46, 48].

The homogeneity of variance was checked for the significance level α=0.05. In the case of lack of homogeneity of variance for all the equation terms, searching for the function of the research object is impossible. It can then be stated that for the assumed significance level α there are no statistical dependencies of input factors on the tested output feature. If it is necessary to determine statistical dependencies, the significance level should be reduced, for example, to the value α=0.07 or α=0.10.
For each equation, correlation coefficients R [49] were determined, reflecting the variability of the studied feature in the obtained approximating equation. It allows for a clear assessment of the effectiveness of regression fitting models.

Table S1. Dependence of the output parameter as a function of input factors with respect to the correlation coefficien *R*

Absolute value of the correlation coefficient R	Correlation – dependence of an output parameter on an input one
0	variables are not correlated

below 0.1	very small
0.1 - 0.3	small
0.3 - 0.5	moderate
0.5 - 0.7	high
0.7 - 0.9	very high
0.9 - 1	almost certain

The correlation coefficient is a numerical measure that indicates the degree to which two or more variables are interdependent [45, 49, 50]. There are many different formulas known as correlation coefficients. Most of them are normalized to take values from −1 (complete negative correlation), through 0 (no correlation) to +1 (complete positive correlation) [41, 49]. Positive correlation is characterized by the fact that when the value of one variable shows an increasing tendency, the value of the other variable also shows an increasing tendency. Negative correlation is characterized by the fact that when the value of one variable shows an increasing tendency, the tendency of the other variable is decreasing [41, 49]. Table S1 presents the relationships between features expressed according to the Stanisz scale. They present the values of correlation coefficients in relation to the relationships of output parameters as a function of input factors.

In addition, the coefficient of determination R^2 and the adjusted coefficient of determination R2-adj were determined for each equation. The values of the R^2 coefficient are a statistical measure of how close the predicted data are to the fitted regression line [39, 47, 49]. The value of the R^2 - adj coefficient can be calculated based on the equation:

$$R^2 - adj = 1 - \frac{N-1}{N-K}(1 - R^2)$$

where:
N- total number of experiments;
K- number of coefficients in the regression equation.

Attempts to determine the dependency function model for the parameters S_p, S_z, S_{sk} or S_{ku} do not allow for determining the models for the assumed confidence level α=0.05. However, the paper presents response graphs for the collected data in Table S2-S6.

Table S2. Results of ANOVA – S_p analysis for vibratory work hardening (without elimination).

	Sum of squares (SS)	Number of degrees of freedom	Mean square	F	p
Model					
t	0.81	1	0.81	2.70	0.176
t^2	0.39	1	0.39	1.29	0.319
f	0.92	1	0.92	3.04	0.156
f^2	0.28	1	0.28	0.93	0.389
tf	1.08	1	1.08	3.59	0.131
Error	1.20	4	0.30		
Total SS	4.49	9			

Table S3. Results of ANOVA – S_z analysis for vibratory work hardening (without elimination).

	Sum of squares (SS)	Number of degrees of freedom	Mean square	F	p
Model	0				
t	4.40	1	4.40	2.24	0.209
t^2	5.12	1	5.12	2.60	0.182
f	6.22	1	6.22	3.16	0.150
f^2	0.90	1	0.90	0.46	0.535
tf	0.18	1	0.18	0.09	0.780
Error	7.88	4	7.88		
Total SS	23.80	9	23.80		

Table S4. Results of ANOVA – S_{ku} analysis for vibratory work hardening (without elimination).

	Sum of squares (SS)	Number of degrees of freedom	Mean square	F	p
Model					
t	0.01	1	0.01	0.00	0.993
t^2	61.70	1	61.70	1.03	0.368
f	134.29	1	134.29	2.23	0.209
f^2	21.42	1	21.42	0.36	0.583
tf	41.47	1	41.47	0.69	0.453
Error	240.39	4	240.39		
Total SS	479.98	9	479.98		

Table S5. Results of ANOVA – S_{sk} analysis for vibratory work hardening (without elimination).

	Sum of squares (SS)	Number of degrees of freedom	Mean square	F	p
Model					
t	0.21	1	0.21	0.22	0.663
t^2	0.83	1	0.83	0.88	0.402
f	0.53	1	0.53	0.56	0.497
f^2	0.34	1	0.34	0.36	0.580
tf	5.18	1	5.18	5.47	0.079
Error	3.78	4	3.78		
Total SS	10.58	9	10.58		

As can be observed, the values of the parameter p in Tables S2-S5 assumed in the vast majority of cases values significantly higher than 0.05. Therefore, in accordance with the data analysis methodology discussed earlier, these terms cannot be included in determining the equations of the RSM response surface for the assumed level of significance α=0.05. The equations describing the influence of t, t^2, f, f^2 and the interaction of factors $t*f$ on S_p, S_z, S_{ku} and S_{sk} are presented in Table S6.

Table S6. Regression equations of vibration hardening treatment indices for brass developed (without elimination).

The regression equation	R^2	R^2-adj
$Sp = 17.84 - 0.144t + 0.0003t^2 - 0.61f + 0.006f^2 + 0.003tf$	0.73	0.40
$Sz = 29.10 - 0.174t + 0.0012t^2 - 0.98f + 0.010f^2 + 0.001tf$	0.67	0.26
$Sku = 85.74 + 0.269t + 0.0041t^2 - 3.79f + 0.049f^2 - 0.016tf$	0.50	0.00
$Ssk = -2.01 - 0.189t - 0.0004t^2 + 0.28f - 0.006f^2 + 0.006tf$	0.64	0.19

The determined R2-Adj values for individual mathematical models in Table S6 show values below 0.5 or even close to 0. This indicates a very small or complete lack of correlation when using the number of variables in the model.
